# Enhanced external quantum efficiency in GaN-based vertical-type light-emitting diodes by localized surface plasmons

**DOI:** 10.1038/srep22659

**Published:** 2016-03-03

**Authors:** Yung-Chi Yao, Jung-Min Hwang, Zu-Po Yang, Jing-Yu Haung, Chia-Ching Lin, Wei-Chen Shen, Chun-Yang Chou, Mei-Tan Wang, Chun-Ying Huang, Ching-Yu Chen, Meng-Tsan Tsai, Tzu-Neng Lin, Ji-Lin Shen, Ya-Ju Lee

**Affiliations:** 1 Institute of Electro-Optical Science and Technology, National Taiwan Normal University, 88, Sec. 4, Ting-Chou Rd., Taipei, 116, Taiwan; 2 Advanced Lighting Technology Department, Green Energy and Environment Research Laboratories, Industrial Technology Research Institute (ITRI), Hsinchu 310, Taiwan; 3 Institute of Photonic System, National Chiao Tung University, 301, Gaofa 3rd Road, Tainan, 711, Taiwan; 4 Institute of Electronics Engineering, National Taiwan University, Taipei 106, Taiwan; 5 Daxin Materials Corporation, No. 15, Keyuan 1st Road, Central Taiwan Science Park, Taichung, 407, Taiwan; 6 Graduate Institute of Photonics, National Changhua University of Education, No.1, Jinde Rd., Changhua 500, Taiwan; 7 Department of Electrical Engineering, Chang Gung University, 259, Wen-Hwa 1st Rd., Kwei-Shan Dist., Taoyuan, 33302, Taiwan; 8 Physics Department, Chung Yuan Christian University, 200, Chung-Pei Rd., Chung Li Dist., Taoyuan, 32023, Taiwan

## Abstract

Enhancement of the external quantum efficiency of a GaN-based vertical-type light emitting diode (VLED) through the coupling of localized surface plasmon (LSP) resonance with the wave-guided mode light is studied. To achieve this experimentally, Ag nanoparticles (NPs), as the LSP resonant source, are drop-casted on the most top layer of waveguide channel, which is composed of hydrothermally synthesized ZnO nanorods capped on the top of GaN-based VLED. Enhanced light-output power and external quantum efficiency are observed, and the amount of enhancement remains steady with the increase of the injected currents. To understand the observations theoretically, the absorption spectra and the electric field distributions of the VLED with and without Ag NPs decorated on ZnO NRs are determined using the finite-difference time-domain (*FDTD*) method. The results prove that the observation of enhancement of the external quantum efficiency can be attributed to the creation of an extra escape channel for trapped light due to the coupling of the LSP with wave-guided mode light, by which the energy of wave-guided mode light can be transferred to the efficient light scattering center of the LSP.

GaN-based light-emitting diodes (LEDs) are revolutionizing the way we illuminate the world. Due to their unique properties, such as low energy consumption, high power emission, and long device lifetime, GaN-based LEDs have been widely applied in traffic signaling, displays, and general lighting. However, light trapping in the GaN (*n*_*GaN*_~ 2.4) due to total internal reflection (TIR) from the sapphire substrates (*n*_*sapphire*_~1.8) limits the light extraction efficiency of traditional GaN-based lateral-type LEDs^1^,^2^,^3^. Recently, GaN-based vertical-type light-emitting diodes (VLEDs) have been developed; such VLEDS promise to alleviate the issue of light trapping. After the reflective metallization bonding and sapphire laser-liftoff processes, the exposed n-side-up surfaces were further textured (or roughened) to frustrate TIR, achieving as high as ~75% of estimated value of light extraction efficiency for a GaN-based VLED^4^,^5^. Meanwhile, the use of one-dimensional (1-D) ZnO nanorods (NRs) synthesized by the hydrothermal method have also been proposed to reduce light trapping^6^,^7^,^8^,^9^,^10^,^11^. The use of ZnO NRs provides another approach to circumvent the costly passivation or tedious photolithography generally required for producing a textured surface in GaN-based LEDs. The typical dimensions of such ZnO NRs synthesized by the hydrothermal method is smaller than the emission wavelength of blue light, so the Rayleigh and Mie scattering can be neglected, and the porous ZnO NRs can be considered as an optically homogeneous film with an effective refractive index ranging between 1 < *n*_*ZnO*_ < *n*_*ZnO (solid film)*_. The synthesized ZnO NRs function as an anti-reflection layer on top of the LED, reducing the Fresnel reflection (*R*) and/or increasing the optical transmission (*T*) at the GaN/air interface. According to electromagnetic theory, for a simple situation of normal incidence and the law of conservation of energy such that *R* + *T* = 1, the optical transmission of the GaN-based LED with synthesized ZnO NRs on top can be expressed as 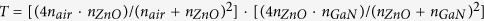, which is slightly larger than that of the bare LED defined as 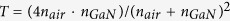. For example, by using above equations, the estimated value of optical transmission is slightly increased from *T* = 82% for a bare LED device to *T* = 93% for a LED capped with a synthesized ZnO NRs layer that has effective refractive index of *n*_*ZnO*_ ~1.49 (corresponding to a porosity of 40%, based on effective medium theory). Although this antireflection layer of ZnO NRs can slightly increase the transmission of emitted light, it cannot increase the critical angle of a bare LED, i.e., the antireflection function of ZnO NRs is only for the small amount of emitted light within the escape cone. As a result, the amount of enhanced light extraction efficiency attributed to the ZnO NRs is indistinct, diminishing the impact of ZnO NRs on the luminescence efficiency of GaN-based LEDs.

Alternatively, the localized surface plasmons (LSPs) in noble metal nanoparticles (NPs) are increasingly studied due to its ability to enhance the photoluminescence (PL) or electroluminescence (EL) intensity of the GaN-based LEDs^12^,^13^,^14^,^15^,^16^,^17^,^18^. The LSP can couple with the exciton in the multiple quantum wells (MQWs) through its extended electromagnetic field in the near-field range, thereby significantly increasing the internal quantum efficiency of a LED^19^,^20^,^21^,^22^. However, the effective transfer of energy between excitons in MQWs and LSPs in NPs through LSP-exciton coupling has a limited distance of approximately several tens of nanometers^23^,^24^. Nevertheless, even for a light emitter that is placed away from the penetration depth of the LSP field, similar energy transfer through LSP-photon coupling remains, as long as the frequency of the photon from the light emitter is coincident with the LSP resonance frequency of NPs^14^,^25^,^26^. The excited LSPs subsequently radiatively decay by emitting photons; hence, the NPs can be conceived as light scattering centers. However, the enhancement of the PL/EL intensity through such energy transfer associated with the LSP-photon coupling effect is rarely discussed for LEDs with NPs located relatively away from the light emitting source.

In this study, the two above-described strategies were combined in the device design of GaN-based VLEDs, and the LSP-photon coupling was demonstrated by drop-casting Ag NPs into the synthesized ZnO NRs to enhance the external quantum efficiency of the LED. Figure 1(a,b) illustrate the ray propagation in the GaN-based VLEDs without (LED *I*) and with (LED *II*) synthesized ZnO NRs on the top surface, respectively. Figure 1(c) shows the ray propagation in the proposed device (LED *III*) with additional Ag NPs decorated on the synthesized ZnO NRs. In Fig. 1(a), the planar surface of LED *I* results in light trapping in the form of wave-guided modes, bouncing up and down in between the metal reflector and the air (blue dashed line). Although an optically homogeneous film of ZnO NRs was synthesized on the top of LED *II* in Fig. 1(b), the majority of the emitted rays still propagate laterally entirely due to the invariance of the light escape cone and the insufficient amount of enhanced optical transmission compared to the case of LED *I*. Such wave-guided modes can be further extracted to increase the external quantum efficiency of the LED by using the LSP resonance coupling effect, which amplifies light intensity in ZnO NRs region via Ag NPs, as shown in Fig. 1 (c). In LED *III*, the synthesized ZnO NRs retain their original function as an anti-reflection coating (ARC) layer to increase the optical transmission for the LED’s luminescence. In addition, with the decoration of Ag NPs inside the synthesized ZnO NRs, the interactions between the trapped photons and the Ag NPs will result in a LSP resonance coupling effect, by which the energy of the wave-guided mode (trapped) light is able to be transferred into the LSPs for efficient light scattering (or re-emission), thereby increasing the external quantum efficiency of the LED.

Herein, we describe the results of theoretical and experimental studies on the enhancement of the external quantum efficiency of GaN-based VLEDs due to the effect of Ag NPs decorated on the synthesized ZnO NRs. Although the dipole source of the light emitter is positioned beyond the electric field of the LSP of the Ag NPs, numerical simulations based on the finite-difference time-domain (*FDTD*) method reveal an effective resonance coupling between the LSPs and the emitted photons. We also experimentally demonstrate a clear increase of the external quantum efficiency of ~22% in our treatment sample. In addition, the synthesis of ZnO NRs and the drop-casting of Ag NPs barely affect the electrical characteristics of the LED. This rational engineering approach of combining the physical phenomena of two different nanostructured materials may provide a promising method for producing high efficiency GaN-based VLEDs.

## Results

**Figure 2:**
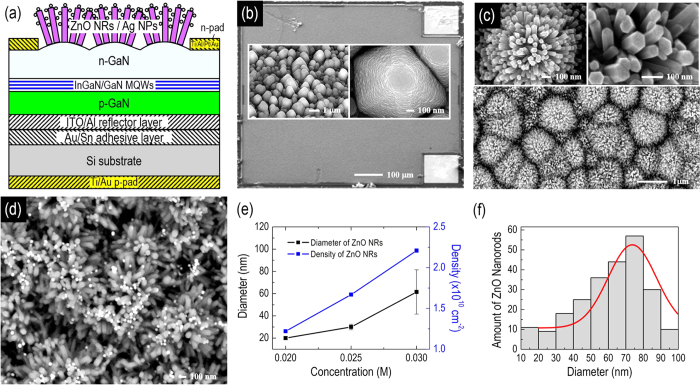
(**a**) Schematic configuration of a GaN-based vertical-type LED (VLED) with Ag NPs decorated on the ZnO NRs that are synthesized on the top surface of the LED. (**b**) Top-view SEM image of the as-fabricated GaN-based VLED. Inset: enlarged images of the roughened features of hexagonal cones on the top surface that enable improved light extraction. (**c**) Top-view SEM images of the GaN-based VLED with the synthesized ZnO NRs. Inset: enlarged SEM images of synthesized ZnO NRs exhibit the typical crystallography of hexagonal structure. (**d**) Top-view SEM image of the GaN-based VLED with the Ag NPs decorated on the synthesized ZnO NRs. (**e**) Distributions of density and diameter as a function of the concentration of the mixture solution for the synthesis of ZnO NRs. (**f**) A histogram of the size distribution of the synthesized ZnO NRs obtained at the solution concentration of 0.03 M.

Figure 2(a) shows the schematic configuration of our proposed GaN-based VLED with Ag NPs decorated on the ZnO NRs that were synthesized on the LED’s top surface using the hydrothermal method. Figure 2(b) shows the top-view scanning electron microscope (SEM) image of our fabricated GaN-based VLED. Hexagonal cones are clearly observed on the N-face of the n-GaN surface after wet etching [Inserts of Fig. 2(b)]. The LED device was then subjected to the hydrothermal process for the growth of ZnO NRs. The randomly aligned ZnO NR arrays were then grown to conformally cover the hexagonal cones, as shown in the SEM images in Fig. 2(c). The ZnO NRs exhibit the typical crystallography of hexagonal structure, which is compatible to the wurtzite crystal structure of the underlying GaN layer. The distributions of density and diameter of the ZnO NRs monotonically increase with the increasing concentration of zinc nitrate hexahydrate [Fig. 2(e)], and an estimated distributed density of the synthesized ZnO NRs of ~ 2.2 × 10^10^ cm^2^ was determined for the solution concentration of 0.03 M. Figure 2(f) shows a statistical analysis of the diameters of 240 ZRs observed in the SEM images, indicating the majority of the nanorods exhibit an average diameter of 60 nm with a standard deviation of ±20 nm, corresponding to an uniformity of ~ 30%. The thickness of ZnO NRs, controllable by the duration time of the synthesis process, is approximately 300 nm in this study. The represented top-view SEM image of LED *III* is shown in Fig. 2(d). The diameter of Ag NPs is approximately 60 ± 5 nm, and the bright spots representing the locations of the Ag NPs are clearly observed.

**Figure 3:**
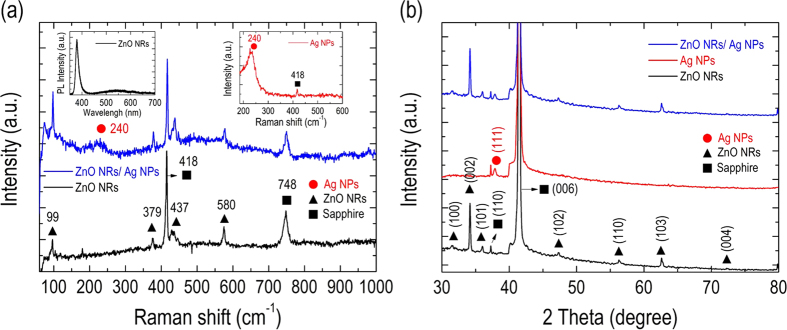
(**a**) Raman spectra of the samples with (blue line) and without (black line) drop-casted Ag NPs decorated on the synthesized ZnO NRs. Inset (upper-right corner): Raman spectrum of bare Ag NPs drop-casting on the sapphire substrate. Inset (upper-left corner): PL spectrum of ZnO NRs synthesized on the sapphire substrate.(**b**) XRD patterns of the samples with (blue line) and without (black line) Ag NPs decorated on the synthesized ZnO NRs; the XRD pattern of bare Ag NPs (red line) drop-casted on the sapphire substrate is also plotted. The diffraction peaks corresponding to the (110) and (006) planes of the sapphire substrate are also marked (as square) in the figure.

Figure 3(a) shows the micro-Raman spectra of the samples with (blue line) and without (black line) Ag NPs decorated on the synthesized ZnO NRs; the Raman spectra are excited by a 532-nm diode-pumped solid-state laser. The micro-Raman spectrum of the bare Ag NPs directly drop-casted onto the sapphire substrate is also shown in Fig. 3(a) for comparison (upper-right corner). Accordingly, the Raman spectrum confirms the hexagonal wurtzite structure of our synthesized NRs. Two distinctive peaks assigned as non-polar E_2_ (high) and E_2_ (low) phonon modes are clearly identified at 437 cm^−1^ and 99 cm^−1^, respectively. In addition, the polar A_1_ transverse A_1_(TO) and E_1_ longitudinal E_1_ (LO) optical modes are observed at 379 cm^−1^ and 580 cm^−1^, respectively, which again suggests the synthesized ZnO NRs are of high crystalline quality. The positions of the Raman scattering peaks of the sapphire substrate are also marked at 418 cm^−1^ and 748 cm^−1^ for the identification purposes. Note that our synthesized ZnO NRs are not subjected to any annealing process, thereby avoiding any possible thermal-budget issues on the highly crystalline GaN VLED, while achieving an acceptable high crystalline quality in the synthesized ZnO NRs. The Raman spectrum of the bare Ag NPs on the sapphire substrate exhibits a considerable band at 240 cm^−1^, which is attributed to the stretching vibrations of the Ag-N^27^ and Ag-O bonds^28^, suggesting the possible formation of chemical bonds with the drop-casting of the Ag NPs. For the Raman spectrum for the sample with Ag NPs decorated on the synthesized ZnO NRs, a relatively weak peak attributed to Ag NPs can still be identified at 240 cm^−1^, implying a successful blending of Ag NPs into the synthesized ZnO NRs via the drop-casting method. To further examine the crystalline quality of our synthesized ZnO NRs, we conducted the photoluminescence (PL) measurements by using a 325 nm He-Cd continuous-wave laser with a spot size of ~100 μm as an excitation source to study ZnO NRs grown by the exact same synthesized condition as described in the Methods section, except for the use of a sapphire substrate. The inset (upper-left corner) of Fig. 3(a) shows the PL spectrum of the ZnO NRs synthesized on the sapphire substrate. Accordingly, a sharp and dominant UV emission centered at λ = 380 nm with FWHM of 30 nm was clearly observed, corresponding to the near-band-edge (NBE) transition of ZnO NRs. A very weak and broad luminescence attributable to the defect-related transitions of ZnO NRs could be observed in wavelength region from λ = 530 nm to λ = 700 nm. The above-described observation on PL spectra indicates relatively low defect and impurity concentrations in the synthesized ZnO NRs, in good agreement with the measured micro-Raman spectra of the ZnO NRs. Most importantly, the result suggests that the use of ZnO NRs on the top surfaces of LED *I* (with emission wavelength of λ = 450 nm) induces a minimal amount of undesired optical absorption associated with the defect transitions of ZnO NRs; such defects would inevitably decrease the light-output power of the LED if there were many of them in the ZnO NRs.

Figure 3(b) shows the X-ray diffraction (XRD) patterns of the samples with (blue line) and without (black line) Ag NPs decorated on the synthesized ZnO NRs. Similarly, the XRD pattern of bare Ag NPs directly drop-casted onto a sapphire substrate is also plotted in the figure (red line) for comparison. The diffraction peaks of the synthesized ZnO NRs are well defined and display the typical hexagonal wurtzite structure with preferred orientations at 2θ = 31.62°, 34.16°, 35.99°, 47.26°, 56.29°, 62.58°, and 72.28°, which corresponds to the (100), (002), (101), (102), (110), (103), and (104) planes, respectively. The XRD patterns further validate the high crystalline quality of our synthesized ZnO NRs, as concluded by the Raman scattering spectra discussed above. A diffraction peak at 2θ = 38.12° attributed to (111) plane of face-centered cubic crystal structure of metallic silver is clearly observed on the bare Ag NPs, and an additional but the same diffraction peak of (111) plane can also be identified for the sample with the Ag NPs decorated on the synthesized ZnO NRs. Again, the result demonstrates that the drop-casting Ag NPs was indeed located in the synthesized ZnO NRs.

## Discussion

**Figure 4:**
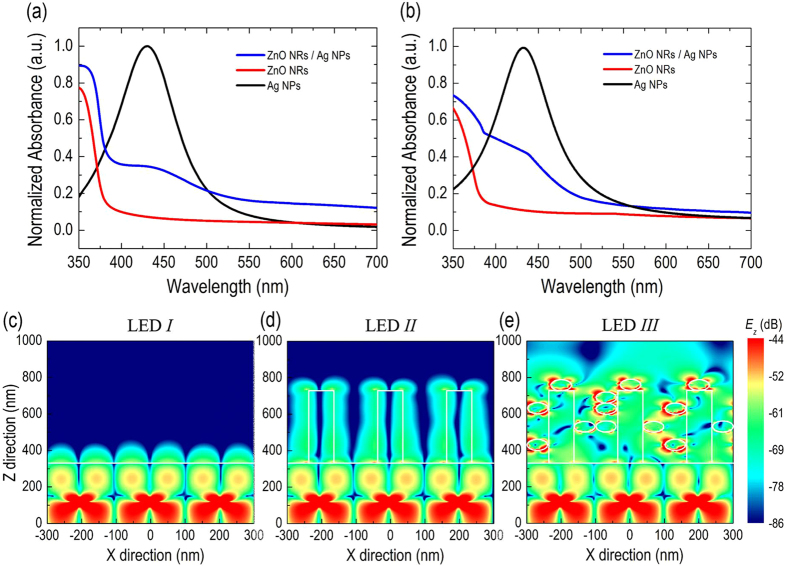
(**a**) Measured, and (**b**) simulated absorption spectra of the synthesized ZnO NRs on the sapphire substrate decorating with (blue line) and without (red line) Ag NPs. The absorption spectra of pure Ag NPs (black line) drop-casted on the sapphire substrate are also plotted in both figures. *FDTD* simulations of the electric field distribution (*E*_*Z*_) for (**c**) LED *I*, (**d**) LED *II*, and (**e**) LED *III*.

To validate the LSP-photon resonance coupling effect to observe the electric field profile of LSPs in the vicinity of Ag NPs decorated on synthesized ZnO NRs, we performed a finite-difference time-domain (*FDTD*) simulation. Figure 4(a) displays the normalized absorption spectra derived from the measured transmittance and reflectance spectra for the ZnO NRs synthesized on the sapphire substrate decorated with (blue line) and without (red line) Ag NPs. The absorption spectrum of pure Ag NPs (black line) drop-casting on the sapphire substrate is also measured and plotted in the figure. The bare ZnO NRs exhibits the typical high absorption behavior in the UV-light region with a sharp cutoff-edge at the incident wavelength of λ = 378 nm, which corresponds to the energy bandgap of ZnO NRs. For the ZnO NRs decorated with Ag NPs, a clear and broad absorption band with the peak wavelength of λ = 433 nm is observed; this absorption band is attributed to the strong LSP resonance coupling of the Ag NPs. However, the absorption peak of pure Ag NPs is slightly blue-shifted to λ = 430 nm, probably due to the different refractive index of the surrounding medium of Ag NPs that mediates the LSP resonance wavelength^29^.

In addition, although the Ag NPs absorb a part of the emission intensity of the LED (λ = 450 nm) to excite the LSP resonances, we also must consider the light scattering (or re-emission) effect from the excited LSP modes, which determines the LSP-enhanced characteristics of the LED. In our situation, the light scattering (or re-emission) appears to dominate over the absorption in the excited LSP resonances, leading to the enhanced external quantum efficiency of the LED. By only considering the absorption spectrum (summation of light absorption and light scattering) of Ag NPs, it is difficult to quantitatively identify these two competing factors between optical absorption and light scattering (or re-emission) in the excited LSP resonances. Thus, further studies are necessary to understand and determine the individual value of absorption and scattering and their respective influences on the enhanced external quantum efficiency of the LED. Figure 4(b) shows the calculated absorption spectra of the same samples obtained by using *FDTD* simulations. Basically, the simulated absorption spectra exhibit a very similar profile to those of experimental measurements. A new broad absorption band attributed to the LSP effect of Ag NPs is again observed on the ZnO NRs decorated with Ag NPs, in qualitative agreement with the experimental results, thus confirming the reliability of our numerical simulations of *FDTD*.

To clarify the effect of the LSP resonance of the Ag NPs on the extraction of the wave-guided optical modes (which propagates along the xy-plane) of the LED, we simulate and observe the distributed electrical fields in the z direction for all of the LEDs to minimize the effect of light within the escape cone (with most of the electric field along the xy-plane). Here, the surface of the VLED is assumed as a planar surface to simplify the *FDTD* simulations. Figure 4(c–e) show the *FDTD* simulations of the electric field profile (*E*_*Z*_) for LED *I*, LED *II*, and LED *III*, respectively. As plotted in Fig. 4(c), the oscillating dipole source located in the MQWs radiates isotropically; however, most of the radiation is confined in the GaN layer because of the TIR effect and is restricted to propagate along the xy plane of the LED. Consequently, *E*_*Z*_ will decay exponentially when it extends into the GaN/air interface as an evanescence wave, in agreement with the fundamental description of wave-guided mode light. For LED *II*, as plotted in Fig. 4(d), the synthesized ZnO NRs on the GaN layer would increase the light transmission by directing the radiation of the dipole source along the axial direction of the ZnO NRs. However, the light out-coupling enhancement of wave-guide mode light due to the bare ZnO NRs is not prominent as the exponential decay of the *E*_*Z*_ field at the ZnO/air interface is clearly observed, implying a significant amount of wave-guided mode light still propagates in the xy plane of the ZnO NRs. Most importantly, although the oscillating dipole source of LED *III* is located far away from the Ag NPs, Fig. 4(e) clearly shows that the electric field of Ag NPs is mostly confined near the surface of NPs, indicating a strong resonance coupling through the dipole radiation and the LSP in the Ag NPs. In addition, the electric field of out-coupling light in LED *III* becomes extensively distributed, and its corresponding intensity remarkably exceeds to those of LED *I* and LED *II*. This result clearly illustrates that the LSP-photon coupling via the large separation between the oscillating dipole source and the metal NPs provides an alternative and efficient approach to enhance the external quantum efficiency of a GaN-based VLED. Note that the result of the *FDTD* simulations provides only a general illustration of the optical interactions between the ZnO NRs and the Ag NPs. It is also applicable to a more realistic case that considers the randomly orientated ZnO NRs. In addition, different from the requirement of a specific polarization of incident light (i.e., p-polarization (polarization along the incident plane)) to excite a surface plasmon polariton, there is no polarization requirement to excite the LSP of Ag NPs. For a spherically shaped nanoparticle, there is no preference for the polarization of incident light due to the symmetry of the particle shape, which is consistent with the absorption spectrum shown in Fig. 4(a) as only one LES resonance mode was observed on our Ag NPs. This characteristic of LSP is an advantage in our design because the cone-shaped rough surface of n-GaN and randomly orientated ZnO NRs will depolarize the incident light^30^,^31^. Therefore, according to the characteristic of non-preferred polarization of LSP excitation and depolarization by a rough surface and randomly orientated ZnO NRs, we believe the enhancement is attributed to TE- and TM-mode nearly equally, although we only simulate *E*_*Z*_-field (to clarify the effect of LSP resonance of Ag NPs) in this study.

**Figure 5:**
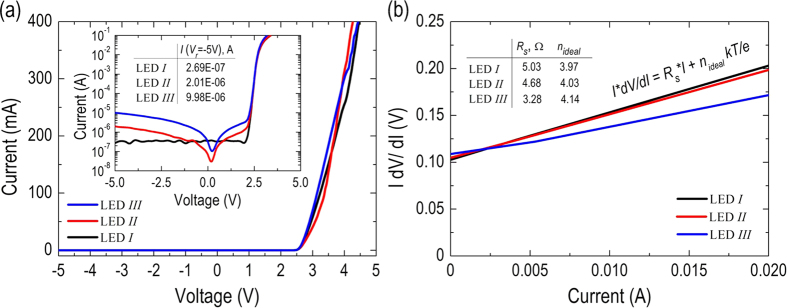
(**a**) Current vs. voltage (*I-V*) behavior of all of the LEDs in linear scales. Inset: re-plotting of the *I-V* curves of all of the LEDs in a semi-log scale. The reverse currents measured at -5 V of all of the LEDs are also summarized and inserted in the figure. (**b**) *I(dV/dI)* versus *I* curves to extract the series resistance (*R*_*S*_) and ideality factors (*n*_*ideal*_) of all of the LEDs. The extracted values of *R*_*S*_ and *n*_*ideal*_ are also summarized and inserted in the figure.

To characterize the electrical properties of all of the fabricated LEDs, current versus voltage (*I-V*) is measured in the dark condition at room temperature. Figure 5(a) shows the measured *I-V* curves of all of the LEDs in linear scales. The inset of Fig. 5(a) re-plots the same *I-V* curves of all of the LEDs in a semi-log scale to assess their leakage currents. Accordingly, the forward voltage at the injection current of *I* = 350 mA are 4.32 V, 4.12 V, and 4.25 V for LED *I*, LED *II*, and LED *III*, respectively, close to that of typical GaN-based high-power LED chips. The result suggests that all of the fabricated LEDs perform good ohmic-contact conditions in both anode and cathode metals. In addition, leakage currents of 0.27 μA, 2.01 μA, and 9.98 μA at −5 V are measured for LED *I*, LED *II*, and LED *III*, respectively, corresponding to an extremely high rectified ratio (±5 V) of 2.10 × 10^6^, 3.24 × 10^5^, and 6.02 × 10^4^, respectively. The slightly higher reverse current measured on LED *III* could be attributed to the fabrication process, such as the drop-casting of Ag NPs or the synthesis of ZnO NRs, that might inevitably cause undesired leakage paths on the sidewalls of the LED device. Nevertheless, the reverse current of 9.98 μA measured on the LED *III* at −5 V corresponds to a shunt resistance of R_p_ ~ 0.48 MΩ, which is generally acceptable while considering the GaN-based VLED with a chip size of 1.0 mm × 1.0 mm. Figure 5(b) plots *I*(*dV*/*dI*) versus *I* to determine the series resistance (*R*_*s*_) and the ideality factor (*n*_*ideal*_) for all of the LEDs according to the differential form of the Shockley diode equation described by *I*(*dV*/*dI*) = *R*_*s*_ *I* + n_*ideal*_ (*kT*/*e*), where *k* and *T* denote to the Boltzmann constant and the ambient temperature, respectively. The extracted values of *R*_*s*_ & *n*_*ideal*_ are *R*_*s*_ = 5.03 Ω & *n*_*ideal*_ = 3.97, *R*_*s*_ = 4.68 Ω & *n*_*ideal*_ = 4.03, and *R*_*s*_ = 3.28 Ω & *n*_*ideal*_ = 4.14 for LED *I*, LED *II*, and LED *III*, respectively. Again, all of extracted values are typical and representative as far as the GaN-based high-power LED chip is concerned. It was also observed that the series resistances of LED *II* and LED *III* are slightly smaller than that of LED *I*, perhaps because the employment of ZnO NRs and Ag NPs promotes the spreading of the injected currents.

**Figure 6:**
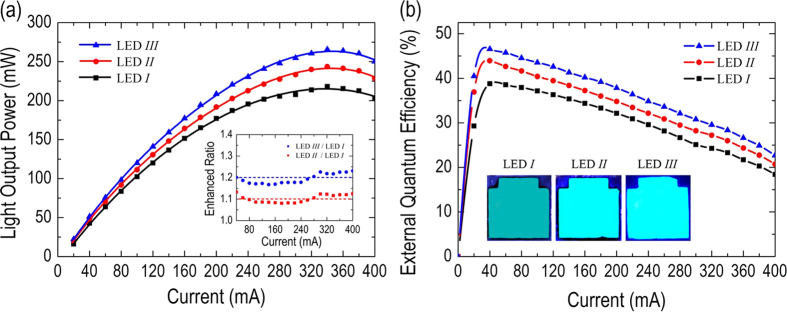
(**a**) Light-output power versus injected current (*L-I* curve) and (**b**) external quantum efficiency (EQE) as a function of injected current for all of the LEDs. Insets: (left) enhanced ratio, defined as the light-output power of the treated sample (LED *II* and LED *III*) divided by that of control sample (LED *I*) vs. injected current; (right) photographs of all of the LEDs taken at *I* = 50 mA.

Finally, we discuss the light-output power characteristics of all of the fabricated LEDs. Figure 6(a) plots the light-output power versus the injected current (*L-I*) for all of the LEDs. The enhanced ratio of light-output power as a function of injected current, which is defined as the light-output power of treatment sample (LED *II* and LED *III*) divided by that of the control sample (LED *I*), is also plotted and inserted in the figure for comparison. The light-output power of all of the LEDs increases sub-linearly with the injected current and is saturated at approximately *I* = 350 mA. Most importantly, the light-output power of LED *III* obviously exceeds that of LED *II* and LED *I*. At *I* = 350 mA, the measured light-output power of LED *I*, LED *II*, and LED *III* is *P* = 216.76 mW, *P* = 242.37 mW and *P* = 264.74 mW, respectively, corresponding to enhanced ratios of light-output power of ~1.12 and ~1.22 for LED *II* and LED *III*, respectively. The enhanced ratios of both treatment LEDs barely changes with the variation of injected current. In addition, because the epitaxial layers and device fabrications of LED *II* and LED *III* herein are identical to those of LED *I*, the synthesis of ZnO NRs and/or the drop-casting of Ag NPs must be primarily responsible for the enhanced external quantum efficiency in LED *II* and LED *III.*

Figure 6(b) shows the external quantum efficiency (EQE) versus injected current for all of the LEDs, and a distinct droop in EQE as the injected current increases is observed. Such EQE reduction with injected current in the GaN-based LEDs is the so-called “efficiency-droop” effect, and its origin has been intensely studied in recent years^32^,^33^. Although the efficiency-droop effect profoundly affects the performance of all of the LEDs, the EQE of LED *III* apparently exceeds those of other LED devices. At *I* = 350 mA, the measured EQE for LED *I*, LED *II*, and LED *III* are 22.50%, 25.15%, and 27.48%, respectively. The EQE of LED *II* exhibits a stable boost of ~ 11.81% over LED *I* under the entire range of injection currents we applied, consistent with the previous discussion that the synthesized ZnO NRs creates better index matching for photon escaping in a manner correlated with a reduction of Fresnel reflection at the GaN/air interface. In LED *III*, the boosted amount on the EQE can be further improved to ~22.12% with the assistance of Ag NPs decorated on synthesized ZnO NRs, and it also remains steady with the increasing of injected currents. Again, besides the reduction of Fresnel reflection due to ZnO NRs, the enhanced EQE in LED *III* is due to the LSP-photon resonance coupling effect that efficiently transfers the energy of wave-guided modes into scattering light (or re-emission) by Ag NPs. The inset in Fig. 6(b) presents a photograph of all of the LEDs taken at *I* = 50 mA. Clearly, the emission intensity of LED *III* is visually brighter than those of other LEDs, validating that our simple strategy of combining different physical fundamentals of two nanostructures for efficient light luminescence via LSP-photon resonance coupling is feasible.

In summary, we demonstrated a viable and highly promising approach to enhance the emission efficiency of GaN-based VLEDs. Densely and vertically aligned ZnO NRs synthesized on the top surface of the LED by hydrothermal method, followed by a direct drop-casting of Ag NPs, exhibits a steady enhancement with the injected currents in the external quantum efficiency (~1.22). The enhanced EQE is mainly attributed to the LSP-photon resonance coupling effect that transfers the energy of wave-guided modes light into LSPs for efficient light scattering (or re-emission), in good agreement with numerical simulations of the electric field distribution of Ag NPs decorated on synthesized ZnO NRs. As a result, even the oscillating dipole source of the LED is located further away from the Ag NPs, a strong resonance coupling through the dipole radiation and the LSP in the Ag NPs can still be expected, providing an alternative and efficient approach for the improvement of the emission efficiency in GaN-based VLEDs.

## Methods

### Fabrication of GaN-based VLEDs

The LED was grown on the sapphire substrate using a low-pressure metal-organic chemical vapor deposition system. The LED structure consists of a low-temperature buffer layer, a 2-μm-thick undoped GaN layer, a 2-μm-thick Si-doped n-type GaN cladding layer, followed by five periods InGaN/GaN MQWs with emitted wavelength of λ = 450 nm, and a 200-nm-thick Mg-doped p-type GaN layer. After subsequent deposition of a 200-nm-thick indium-tin-oxide and a 500-nm-thick Al reflector on top of the LED structure, the LED wafer was flipped and bonded to silicon substrate by Au/Sn metal bonding at T = 350 °C^34^. A KrF excimer laser lift-off process was subsequently used to remove the sapphire substrate. Next, the n-side-up GaN layer exhibiting an *N*-face (000-1) surface was exposed. The LED chip-size of 1 × 1 mm^2^ was patterned and fabricated by photolithography and dry-etching. Ti/Al/Pt/Au and Ti/Au were deposited by the thermal evaporator and patterned by lift-off process to form the n-type and p-type ohmic contacts of the LED, respectively. Dilute potassium hydroxide (KOH) of 80 °C was then used as the etching solution to roughen the top surface of the LED (i.e., LED *I*) for 3 min.

### Synthesis of ZnO nanorods (NRs)

Initially, a 30 nm ZnO seed layer was directly sputtered onto the roughened N-face n-GaN surface, followed by suspending the sample in an aqueous solution of 400 mL of zinc nitrate hexahydrate (Zn(NO_3_)_2_·6 H_2_O, 0.025 mol/L) and 100 mL of hexamethylenetetramine ((CH_2_)_6_·N_4_, 0.025 mol/L) at 90 °C for 60 min in an oven.

### Spreading of Ag nanoparticles (NPs)

The aqueous solution of Ag NPs (Sigma-Aldrich Co.) with the concentrations of 0.02 mg/mL was drop-casted on the sample and then dried in the atmosphere for several minutes (i.e., LED *III*).

### Simulation

For the *FDTD* simulations, the experimental values for the metal permittivity of Ag were used^35^, and the refractive index of GaN and ZnO NRs was set to 2.49 and 1.99, respectively. The diameter of Ag NPs was set to 60 nm, and the synthesized length of ZnO NRs was set to 400 nm. The Ag NPs were randomly placed on ZnO NRs to mimic the situation in the real device. The synthesized ZnO NRs was placed on the top of GaN layer. The oscillating dipole source (λ = 450 nm) was positioned 215 nm underneath the GaN/ZnO NRs interface, corresponding to the location of the MQW region.

### Characterization

Morphologies and crystallization nature of ZnO NRs were characterized using field emission SEM (JEOL, JSM7600F, 10 kV) and XRD (Bruker, D2 PHASER, with Cu-K_a_ radiation). The molecular morphologies of synthesized ZnO NRs corresponding to specific vibrational frequencies of bonds shown in Raman bands were observed by the micro-Raman spectrometer (HORIBA JOBIN YVON, iHR550, equipped with a 532-nm diode-pumped solid state laser). The current-voltage (*I-V*) measurement of the LEDs was performed using a semiconductor parameter analyzer (Agilent, B1500A). The light output power of each LED was measured from the top of the LEDs by using a Si photodiode connected to an optical power meter. The light-emission images of the LEDs were acquired using a microscope equipped with a CCD camera.

## Additional Information

**How to cite this article**: Yao, Y.-C. *et al.* Enhanced external quantum efficiency in GaN-based vertical-type light-emitting diodes by localized surface plasmons. *Sci. Rep.* **6**, 22659; doi: 10.1038/srep22659 (2016).

## Figures and Tables

**Figure 1:**
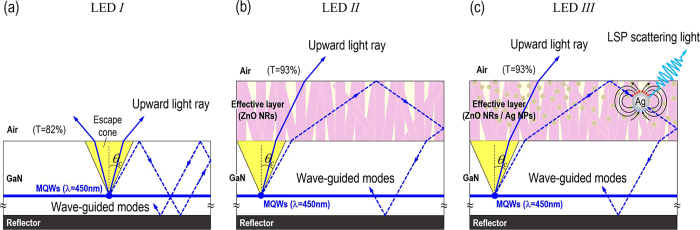
Schematic illustration of light propagating in GaN-based VLEDs (**a**) without and (**b**) with synthesized ZnO NRs on the top surface. (**c**) The ray propagation for the Ag NPs decorated on the ZnO NRs synthesized on the top of GaN-based VLED. The light escape cones defined by the critical angle of TIR [arcsin (n_air_/n_GaN_), marked by yellow regions] are identical for all of the LEDs. The blue dashed lines indicate the wave-guided modes of the emitted light.
